# Coronavirus Disease 2019 Severity and Risk of Subsequent Cardiovascular Events

**DOI:** 10.1093/cid/ciac661

**Published:** 2022-09-05

**Authors:** Timothy L Wiemken, Leah J McGrath, Kathleen M Andersen, Farid Khan, Deepa Malhotra, Tamuno Alfred, Jennifer L Nguyen, Laura Puzniak, Elizabeth Thoburn, Luis Jodar, John M McLaughlin

**Affiliations:** Pfizer Inc, Collegeville, Pennsylvania, USA; Pfizer Inc, Collegeville, Pennsylvania, USA; Pfizer Inc, Collegeville, Pennsylvania, USA; Pfizer Inc, Collegeville, Pennsylvania, USA; Pfizer Inc, Collegeville, Pennsylvania, USA; Pfizer Inc, Collegeville, Pennsylvania, USA; Pfizer Inc, Collegeville, Pennsylvania, USA; Pfizer Inc, Collegeville, Pennsylvania, USA; Pfizer Inc, Collegeville, Pennsylvania, USA; Pfizer Inc, Collegeville, Pennsylvania, USA; Pfizer Inc, Collegeville, Pennsylvania, USA

**Keywords:** PASC, postacute COVID-19 syndrome, SARS-CoV-2, long COVID, post-COVID syndrome

## Abstract

**Background:**

Little is known about the relationship between coronavirus disease 2019 (COVID-19) severity and subsequent risk of experiencing a cardiovascular event (CVE) after COVID-19 recovery. We evaluated this relationship in a large cohort of United States adults.

**Methods:**

Using a claims database, we performed a retrospective cohort study of adults diagnosed with COVID-19 between 1 April 2020 and 31 May 2021. We evaluated the association between COVID-19 severity and risk of CVE >30 days after COVID-19 diagnosis using inverse probability of treatment–weighted competing risks regression. Severity was based on level of care required for COVID-19 treatment: intensive care unit (ICU) admission, non-ICU hospitalization, or outpatient care only.

**Results:**

A total of 1 357 518 COVID-19 patients were included (2% ICU, 3% non-ICU hospitalization, and 95% outpatient only). Compared to outpatients, there was an increased risk of any CVE for patients requiring ICU admission (adjusted hazard ratio [aHR], 1.80 [95% confidence interval {CI}, 1.71–1.89]) or non-ICU hospitalization (aHR, 1.28 [95% CI, 1.24–1.33]). Risk of subsequent hospitalization for CVE was even higher (aHRs, 3.47 [95% CI, 3.20–3.76] for ICU and 1.96 [95% CI, 1.85–2.09] for non-ICU hospitalized vs outpatient only).

**Conclusions:**

COVID-19 patients hospitalized or requiring critical care had a significantly higher risk of experiencing and being hospitalized for post–COVID-19 CVE than patients with milder COVID-19 who were managed solely in the outpatient setting, even after adjusting for differences between these groups. These findings underscore the continued importance of preventing severe acute respiratory syndrome coronavirus 2 infection from progressing to severe illness to reduce potential long-term cardiovascular complications.

## Teaser

COVID-19 patients hospitalized with or without intensive care unit admission had 80% and 28%, respectively, higher risk of post–COVID-19 cardiovascular events and were 2–4 times more likely to be hospitalized for a subsequent cardiovascular event than those requiring outpatient care only.

An estimated 30% of coronavirus disease 2019 (COVID-19) survivors continue to experience an array of symptoms for weeks to months after initial diagnosis [1], suggesting that many individuals have incomplete recovery from acute illness [2–5]. The constellation of postacute COVID-19 syndromes, persistent symptoms, and new ailments are termed postacute sequelae of severe acute respiratory syndrome coronavirus 2 (SARS-CoV-2) infection (PASC), informally known as “long COVID” [6]. Although the most widely recognized PASC syndrome is multisystem inflammatory syndrome in both adults and children (MIS-A and MIS-C, respectively) [7], a multitude of other post–COVID-19 outcomes have been documented, spanning from persistent fatigue [8] and sleep difficulties [4] to type 1 diabetes [9] and neurological manifestations [10]. The incidence of these syndromes varies significantly and appears to be driven by the demographic and clinical characteristics of the patient [11].

The pathophysiology of SARS-CoV-2 infection suggests the ability to produce cardiac damage after infection. Several investigations and reviews suggest that multiple cardiovascular complications such as hypertension, arrhythmia, thromboembolism, acute myocardial infarction, cerebrovascular accident, and others may be more frequent after recovery from COVID-19 [12–24]. Limited data have suggested that more severe disease may lead to a higher probability of cardiovascular complications. However, this has not been widely investigated in the general population and was assessed only as a secondary outcome in 1 study conducted among United States (US) Veterans [12]. Thus, we evaluated the association between COVID-19 severity and the risk of subsequent cardiovascular events (CVEs) among adults in a large, generalizable US population.

## METHODS

### Study Design and Population

We performed a retrospective cohort study of adults (≥18 years of age) using nationwide health insurance claims data from the US HealthVerity Real-Time Insights and Evidence database, including both open- and closed-source claims.

Study eligibility required medical and pharmacy coverage and a diagnosis for COVID-19 (*International Classification of Diseases, Tenth Revision, Clinical Modification* [*ICD-10-CM*] code U07.1) between 1 April 2020 and 31 May 2021, with at least 365 days of continuous medical and pharmacy enrollment before index. The patient’s date of first COVID-19 diagnosis served as the index date. We excluded patients with a documented history of any cardiovascular outcome under analysis or with antithrombotic use 365 days before index, as well as patients who experienced any cardiovascular outcome or died within 30 days after index.

### Outcomes and Follow-up

Prespecified outcomes were selected to replicate major CVE groups as outlined in Xie et al [12]. The primary outcome was the first occurrence of dysrhythmia, ischemic heart disease, thrombotic disorders, cerebrovascular accident (ie, stroke or transient ischemic attack), or other cardiac disorders (eg, heart failure, myocarditis). Secondarily, we considered each CVE group separately and for atherosclerotic, inflammatory, and acute and chronic CVEs. We also repeated all analyses where only CVEs requiring inpatient care were included as an outcome to reduce potential detection biases whereby patients with more severe COVID-19 might have been followed more closely for sequelae thereafter. For persons who remained hospitalized ≥30 days after COVID diagnosis, any CVE that occurred in the inpatient setting, regardless of whether it occurred during the continuation of the initial hospitalization or as a new admission, was counted. All outcomes were identified using *ICD-10-CM* codes (Supplementary Table 1) [25]. Code lists used the COVID-19 Natural History Master Protocol from the US Food and Drug Administration’s Sentinel Initiative [26] and were augmented by expert medical review. Patients were followed starting >30 days after the index until the event of interest. Patients were censored if they disenrolled from the data source or at the end of the study period (31 December 2021).

### Exposure

COVID-19 site of care was defined as the highest level of care experienced over the 30-day window after index as a proxy for COVID-19 disease severity: intensive care unit (ICU) admission, non-ICU hospitalization, or outpatient care only. To classify hospitalizations as “for” COVID-19 rather than “with” COVID-19, the COVID-19 diagnosis was required to be present on admission or the principal admitting diagnosis. ICU status was identified using revenue codes, Healthcare Common Procedure Coding System (HCPCS) codes, or *Current Procedural Terminology, Fourth Edition* (*CPT-4*) codes as used in a previously defined algorithm [27].

### Covariates

Unless otherwise noted, all predefined covariates were assessed 365 days before the index. Demographics included age, sex, region of residence (US Census region categories: Northeast, Midwest, South, West), and insurance provider. The month of COVID-19 diagnosis was used to account for any pandemic-related time trends, including circulating variants. Clinical characteristics were identified using *ICD-10-CM* diagnosis codes, HCPCS codes, and *CPT-4* procedure codes. These included individual variables of the Charlson Comorbidity Index; other measures of poor health, including immunosuppressive conditions, smoking status, obesity, hypertension (defined based on diagnosis or medication usage), prior hospitalization, prior or current residence in a nursing home or skilled nursing facility, impaired functional status (ie, required nursing facility services, at-home medical visit services, supplemental oxygen, or wheelchair use), and measures of preventive health (history of influenza and pneumococcal vaccination and history of at least 1 wellness visit in the year before index). Prior COVID-19 vaccination was assessed using National Drug Codes and *CPT-4* vaccine administration codes. Indicator variables were used in propensity score estimation for missing sex or region variables. The absence of a code for all other covariates, such as comorbid conditions, was considered to be the absence of the condition rather than missing data [28].

### Statistical Analysis

Baseline characteristics were summarized with counts and percentages for categorical variables and means with standard deviations for continuous variables. Multinomial propensity score models were used to calculate stabilized inverse probability of treatment weights (IPTW) for patients across care settings to account for imbalances in baseline characteristics between patients in different care settings. Covariate balance before and after weighting was assessed using standardized mean difference (SMD) as ICU admission or non-ICU hospitalization vs outpatient care only. Variables with residual imbalance (>10% SMD) were included in the final model for further adjustment.

Weighted cumulative incidence curves were calculated to describe the occurrence of each outcome separately over the entire follow-up period. Proportional subdistribution hazard models were used to estimate adjusted hazard ratios (aHRs) and 95% confidence intervals (CIs), accounting for the competing risk of death. Finally, we quantified the amount of independent unmeasured confounding that would have had to be present to change the interpretation of the results using E-values. All analyses were performed in SAS, version 9.4 (SAS Institute, Cary, North Carolina) and R version 3.6.2 (R Foundation for Statistical Computing, Vienna, Austria) software.

We also performed subgroup analyses replicating the primary analyses separately for each of the following age groups: 18–49, 50–64, and ≥65 years. Standard Cox proportional hazards models were used for the 18–49 subgroup due to the low number of deaths experienced in this subgroup. Propensity scores were reestimated in each analysis, and a doubly robust set of covariates was reassessed.

### Conduct and Ethics Statements

The study followed Strengthening the Reporting of Observational Studies in Epidemiology reporting guidelines [29]. This study was deemed exempt from Institutional Review Board (IRB) review pursuant to the terms of the US Department of Health and Human Services’ Policy for Protection of Human Research Subjects at 45 Code of Federal Regulations 46.104(d); category 4 exemption (Sterling IRB, Atlanta, Georgia, waived ethical approval for this work).

## RESULTS

**Figure 1.**
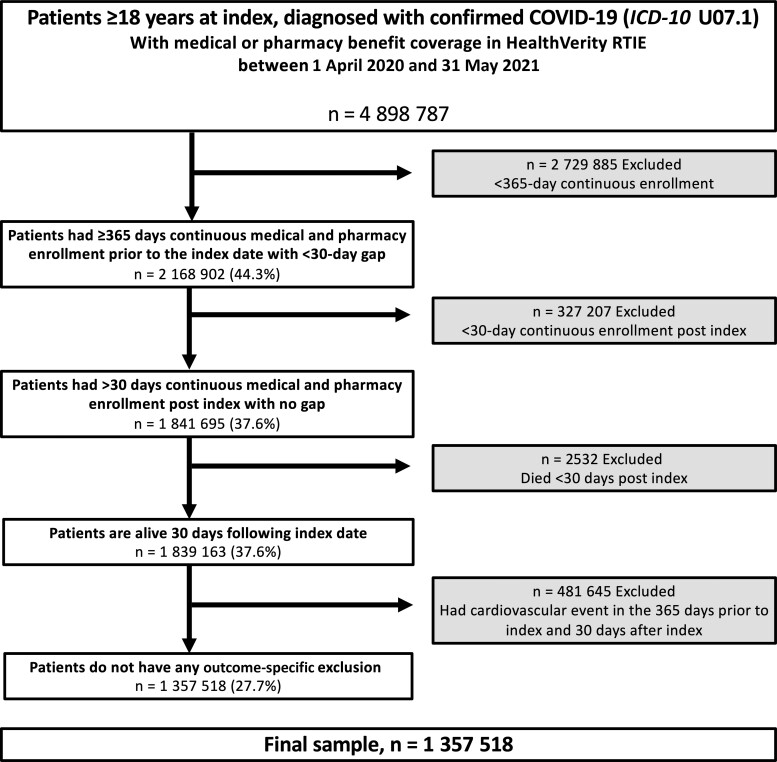
Study flowchart. Abbreviations: COVID-19, coronavirus disease 2019; *ICD-10*, *International Classification of Diseases, Tenth Revision*; RTIE, Real-Time Insights and Evidence.

There were 4 898 787 eligible adults (≥18 years of age) with COVID-19 in the database, of whom 1 357 518/4 898 787 (27.7%) met the selection criteria for final analysis (Figure 1). Most were treated in the outpatient setting only (1 292 064 [95.2%]), 44 385 (3.3%) required hospitalization without ICU admission, and 21 069 (1.6%) were admitted to ICU (Table 1). Outpatients tended to be younger than patients requiring ICU admission and non-ICU hospitalized patients (mean age, 41 years vs 54 and 50 years, respectively) and had a lower prevalence of obesity (20% vs 33% and 30%, respectively), diabetes without complications (13% vs 35% and 27%, respectively), chronic kidney disease or end-stage renal disease (5% vs 16% and 13%, respectively), and hospitalization in the prior year (7% vs 16% and 18%, respectively) (Table 1). Trends were similar when stratified by age group (Supplementary Tables 2–4). Weighted cohorts were well balanced, with no remaining imbalances in the non-ICU hospitalized vs outpatient comparisons. Only a single variable (age) was included in the doubly robust outcome model for the ICU admission group comparison (Supplementary Table 5).

**Table 1.** Characteristics of Coronavirus Disease 2019 Patients Meeting Inclusion Criteria for Analysis (N = 1 357 518)

Characteristic	Outpatient (n = 1 292 064)	Non-ICU Hospitalization (n = 44 385)	ICU Admission (n = 21 069)	Weighted SMD: Non-ICU Hospitalization vs Outpatient	Weighted SMD: ICU Admission vs Outpatient
Age, y, mean (SD)	41 (16)	50 (18)	54 (16)	0.058	0.104
Sex				0.067	0.012
ȃFemale	781 345 (60)	26 793 (60)	10 911 (52)		
ȃMale	504 999 (39)	17 258 (39)	10 050 (48)		
ȃUnknown	5720 (<1)	334 (1)	108 (<1)		
Region				0.021	0.057
ȃNortheast	253 182 (20)	10 088 (23)	3968 (19)		
ȃMidwest	296 182 (23)	9623 (22)	4130 (20)		
ȃSouth	583 955 (45)	19 357 (44)	9873 (47)		
ȃWest	155 522 (12)	5195 (12)	3071 (15)		
ȃOther	3128 (<1)	119 (<1)	24 (<1)		
ȃMissing	95 (<1)	3 (<1)	3 (<1)		
≥1 COVID-19 vaccine dose	13 533 (1)	350 (1)	171 (1)	−0.047	−0.044
Indicators of poor health					
ȃSmoking	118 289 (9)	6369 (14)	2789 (13)	0.024	0.018
ȃObesity	264 017 (20)	13 404 (30)	7033 (33)	0.073	0.090
ȃNursing home residence	13 946 (1)	909 (2)	466 (2)	0.019	0.032
ȃSkilled nursing facility	17 235 (1)	1139 (3)	537 (3)	0.025	0.037
ȃPrior hospitalization	84 842 (7)	7949 (18)	3345 (16)	0.020	0.018
ȃImpaired functional status	36 109 (3)	3415 (8)	1697 (8)	0.026	0.059
Indicator of health-seeking behavior					
ȃPneumococcal vaccine	32 714 (3)	2168 (5)	1299 (6)	0.015	0.026
ȃInfluenza vaccine	433 282 (34)	15 298 (34)	7556 (36)	0.034	0.018
ȃWellness visit	804 083 (62)	25 088 (57)	11 884 (56)	0.018	–0.010
Hypertension	352 584 (27)	19 934 (45)	11 411 (54)	0.045	0.082
Comorbid conditions^a^					
ȃMyocardial infarction	3488 (<1)	249 (1)	133 (1)	0.008	0.010
ȃCongestive heart failure	5589 (<1)	511 (1)	287 (1)	0.005	0.019
ȃPeripheral vascular disease	40 069 (3)	3278 (7)	1939 (9)	0.025	0.028
ȃCerebrovascular disease	17 369 (1)	1290 (3)	666 (3)	0.013	0.029
ȃDementia	15 441 (1)	1552 (3)	734 (3)	0.010	0.023
ȃChronic pulmonary disease	197 317 (15)	9486 (21)	4778 (23)	0.025	0.049
ȃRheumatic disease	28 539 (2)	1502 (3)	807 (4)	0.019	0.023
ȃPeptic ulcer disease	11 087 (1)	729 (2)	353 (2)	0.010	0.012
ȃMild liver disease	77 435 (6)	4409 (10)	2532 (12)	0.029	0.051
ȃDiabetes without complications	168 215 (13)	11 856 (27)	7373 (35)	0.046	0.098
ȃDiabetes with complications	54 980 (4)	5156 (12)	3448 (16)	0.027	0.048
ȃHemiplegia or paraplegia	5304 (<1)	492 (1)	300 (1)	0.009	0.028
ȃAny malignancy	36 265 (3)	2352 (5)	1255 (6)	0.014	0.024
ȃModerate/severe liver disease	2567 (<1)	321 (1)	211 (1)	0.008	0.014
ȃMetastatic solid tumor	4776 (<1)	431 (1)	220 (1)	0.006	0.012
ȃHIV/AIDS	7016 (1)	470 (1)	210 (1)	0.004	0.006
ȃCKD/ESRD	70 571 (5)	5898 (13)	3447 (16)	0.024	0.043
High-risk immunocompromised conditions					
ȃSolid malignancy	167 019 (13)	6526 (15)	3341 (16)	0.008	0.020
ȃHematologic malignancy	3227 (<1)	313 (1)	184 (1)	0.004	0.010
ȃBone marrow transplant	192 (<1)	19 (<1)	12 (<1)	0.002	−0.002
ȃOrgan transplant	2533 (<1)	399 (1)	202 (1)	0.005	0.013
ȃRheumatologic or other inflammatory condition	105 910 (8)	5065 (11)	2691 (13)	0.017	0.034
ȃPrimary immunodeficiency	20 480 (2)	1589 (4)	909 (4)	0.009	0.019
ȃOther immune condition	30 450 (2)	1823 (4)	905 (4)	0.011	0.006
ȃImmunosuppressive medication ≥14 d	78 312 (6)	3616 (8)	2067 (10)	0.021	0.026
ȃAntimetabolite medication ≥ 14 d	8952 (1)	599 (1)	330 (2)	0.011	0.016

Data are presented as No. (%) unless otherwise indicated.

Abbreviations: CKD, chronic kidney disease; COVID-19, coronavirus disease 2019; ESRD, end-stage renal disease; HIV, human immunodeficiency virus; ICU, intensive care unit; SD, standard deviation; SMD, standardized mean difference.

Measured using all-available history before the index date.

**Figure 2.**
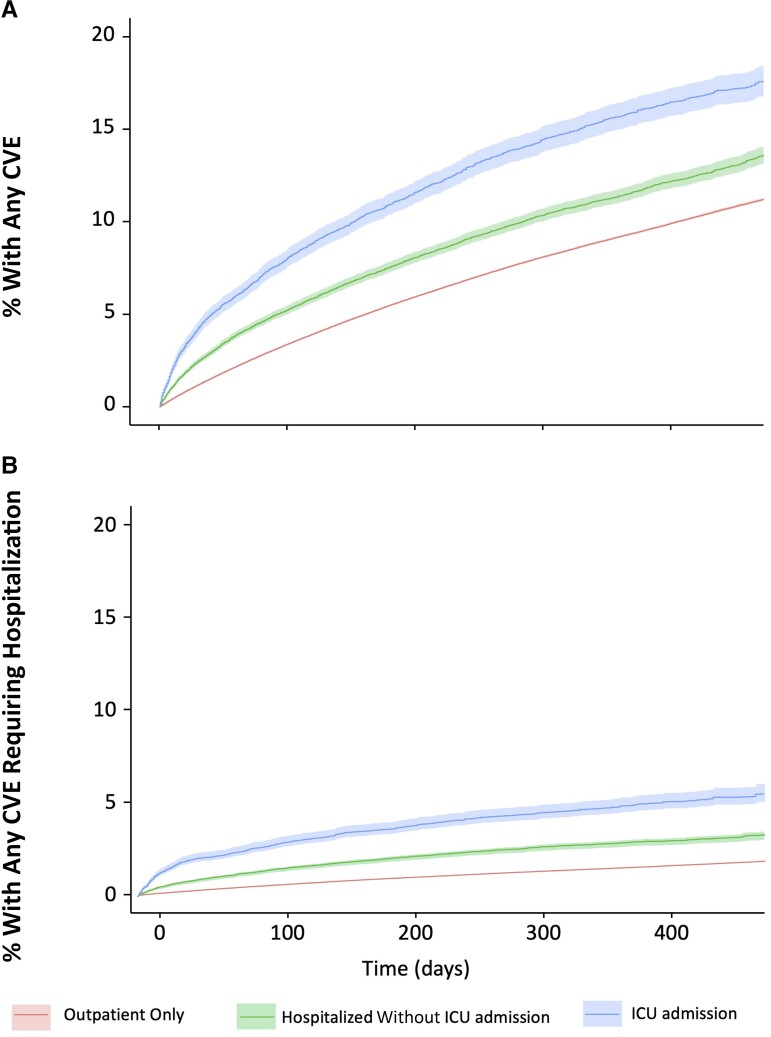
Weighted cumulative incidence of cardiovascular events (CVEs) by level of care required among patients with coronavirus disease 2019. *A*, Any postacute CVE. *B*, Postacute CVE requiring hospital admission. Abbreviation: ICU, intensive care unit.

For each group, the median was 1 inpatient visit in the first 6 months of follow-up (Supplementary Table 6). For outpatient visits, there was an increasing number of outpatient visits with increasing acute COVID-19 severity. We observed a dose-response between the severity of COVID-19 and the incidence of any CVE (Figure 2*A*). This relationship was observed early on and was maintained through the end of the follow-up. At 9 months after COVID-19 diagnosis, the weighted cumulative incidences of any CVE event were 14% among patients requiring ICU care, 10% among non-ICU hospitalized patients, and 8% among outpatients (Figure 2*A* and Supplementary Table 7) and continued to increase throughout follow-up for all 3 groups. Similar trends were observed for each CVE outcome, with dysrhythmia the most commonly occurring outcome and thrombotic disorders the least common (Supplementary Figure 1).

We also observed a dose-response between the severity of COVID-19 and the incidence of any CVE requiring hospitalization (Figure 2*B*). Weighted cumulative incidence of CVEs requiring hospitalization at 9 months after COVID-19 diagnosis was 4%, 2%, and 1% among the 3 groups, respectively (Figure 2*B* and Supplementary Table 8).

**Figure 3.**
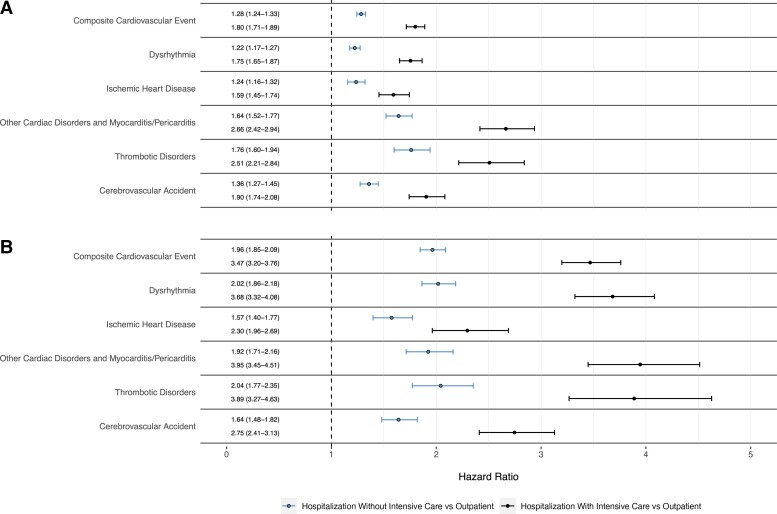
Forest plot of adjusted hazard ratios (aHRs) and 95% confidence intervals (CIs) from competing risks survival analysis evaluating the association between site of care and incident cardiovascular events (CVEs) among patients with coronavirus disease 2019. *A*, Any postacute CVE. *B*, Postacute CVE requiring hospital admission. See Supplementary Tables 7 and 8 for raw values as well as weighted and unweighted aHRs with 95% CIs.

In adjusted competing risk regression models, COVID-19 patients requiring ICU admission (adjusted aHR, 1.80 [95% CI, 1.71–1.89]) and non-ICU hospitalized patients (aHR, 1.28 [95% CI, 1.24–1.33]) had significantly higher risk of CVE compared to COVID-19 patients treated only in the outpatient setting (Figure 3*A* and Supplementary Table 7). E-values for the composite outcome were 1.88 for non-ICU hospitalized and 3.00 for ICU patients (Supplementary Table 9). This effect was even more pronounced when restricting the outcome to CVEs requiring hospitalization (aHR, 3.47 [95% CI, 3.20–3.76]) for ICU patients and aHR, 1.96 [95% CI, 1.85–2.09]) for non-ICU hospitalized patients vs outpatients (Figure 3*B* and Supplementary Table 8). We observed similar trends when categorizing CVEs into atherosclerotic and inflammatory events and acute and chronic events (Supplementary Table 10).

The risk of individual CVEs was also elevated for both groups of hospitalized COVID-19 patients (ie, with or without ICU admission) compared to outpatients, with a larger risk for each CVE among those requiring ICU care. Compared to outpatients, risks of individual cardiac conditions were increased by 59%–166% for COVID-19 patients admitted to ICU and 22%–76% for non-ICU hospitalized patients (Figure 3*A* and Supplementary Table 7). The largest effect sizes were found among patients admitted to the ICU for the risk of other cardiovascular disorders (aHR, 2.66 [95% CI, 2.42–2.94]) and thrombotic disorders (aHR, 2.51 [95% CI, 2.21–2.84]).

When stratified by age group, among adults requiring ICU admission for COVID-19, the risk of any CVE was higher among patients 18–49 years of age (aHR, 1.92 [95% CI, 1.79–2.05]) compared to those aged 50–64 years (aHR, 1.64 [95% CI, 1.55–1.75]) and ≥65 years (aHR, 1.36 [95% CI, 1.26–1.48]). These trends were consistent across individual CVEs except for dysrhythmia, where middle-aged adults had the higher risk (Supplementary Tables 11–13).

Similarly, when stratified by age group, among adults requiring ICU admission for COVID-19, the risk of any CVE requiring a subsequent hospitalization was higher among patients 18–49 years of age (aHR, 4.04 [95% CI, 3.56–4.59]) compared to those 50–64 (aHR, 2.87 [95% CI, 2.58–3.20]) and ≥65 years (aHR, 2.23 [95% CI, 1.95–2.55]) (Supplementary Tables 14–16). These trends were generally consistent across individual CVEs and among non-ICU hospitalized adults, except for thrombotic disorders and cerebrovascular accidents, where middle-aged adults had a higher risk.

## DISCUSSION

Despite broad public health efforts, the large and growing number of individuals infected with SARS-CoV-2 [30, 31] is concerning in terms of how many may experience long-term sequelae, such as CVEs, in the months and years ahead. Our study suggests that increasing the severity of COVID-19 illness increases the risk of developing a subsequent, nonacute CVE among individuals without a history of cardiac illness in the prior year, independent of the many factors included in the IPTW analysis. Specifically, compared to patients who had COVID-19 that required outpatient care only, those who required ICU admission were 80% more likely, and those who required non-ICU hospitalization 28% more likely, to experience a CVE >30 days after the initial COVID-19 episode. Compared to outpatients, these same 2 groups were 247% and 96% more likely, respectively, to be hospitalized for a CVE after COVID-19 illness. This dose-response relationship persisted after adjustment for many potentially confounding patient demographic and clinical characteristics (eg, age, month of COVID-19 diagnosis, obesity, smoking, immunocompromising status, and other relevant comorbidities). Standardized mean differences and E-values provided quantitative evidence of the risk of bias from residual confounding, and suggested that our results are unlikely to be entirely explained by patient characteristics rather than solely COVID-19 severity.

While the incidence of cardiovascular sequelae is lower in younger adults than older adults, we saw larger relative hazards for the youngest age group (18–49 years). Older adults are a well-described group at higher risk of severe COVID-19, yet these results also underscore the importance of mitigation measures among younger adults. Similarly, the more severe outcomes, such as thrombotic events and cerebrovascular accidents, while rare overall, occur substantially more often among the more severe COVID-19 cases than those managed in the outpatient setting. These findings reiterate the importance of vaccination for preventing SARS-CoV-2 infection and reducing its severity—as results from our study suggest that subsequent CVEs appear to be linked to more severe COVID-19 illness. Likewise, these results support the prompt treatment of acute COVID-19 illness with antivirals to minimize severe disease from developing to help reduce the risk of potentially life-threatening post–COVID-19 cardiovascular events.

Our results are consistent with Xie et al, who reported approximately 1.5–2.0 times higher risk of various CVEs after SARS-CoV-2 infection in a US Veterans Affairs population. These results also showed a dose-response with increasing severity of disease [12]. Similarly, Jovanoski and colleagues reported a higher risk of CVEs after COVID-19, which increased with increasing disease severity in a patient cohort from the Optum electronic health record database [32]. Recently, myocarditis and pericarditis have been found to be substantially higher in patients who have recovered from COVID-19 [33], which is also consistent with our secondary analyses.

Interestingly, increased cardiovascular risk after acute infection may not be unique to COVID-19. Although few studies have evaluated long-term cardiovascular risks, several have linked other severe infections such as bacteremia, influenza, and pneumonia with acute CVEs [34–43]. For example, Ou and colleagues reported a 22%–65% increased risk of CVEs in sepsis survivors persisting for up to 5 years after hospital discharge. However, this was not statistically significant after multivariable modeling [44]. Corrales-Medina et al suggested there could be a 50%–60% higher risk of cardiovascular disease after hospitalization due to pneumonia 5–10 years after discharge [45]. Yende and colleagues identified a 10% increased risk in CVEs in sepsis survivors compared with matched hospitalized or ICU control subjects [46].

There is uncertainty about the biological mechanisms behind the apparent increased risk of CVEs following SARS-CoV-2 infection. SARS-CoV-2 infects cardiac myocytes through the ACE2 receptor and may remain persistent, invoking chronic inflammatory responses and subsequent tissue damage or fibrosis [47]. Another mechanism is thought to be an autoimmune response to cardiac antigens resulting in delayed damage to cardiac tissues [48, 49]. These autoantibodies may target various systems, including platelets, phospholipids, and endothelial cells, and may activate neutrophils or promote thrombosis [23]. Further, anti-heart antibodies have been shown to correlate with cardiovascular manifestation in COVID-19 patients and, importantly, correlate with the severity of illness [50]. Other potential mechanisms of cardiovascular damage include direct viral toxicity leading to long-term cardiac damage or thrombosis in vasculitis, either of which may result in immediate or delayed risk to cardiac health [47]. Future studies should attempt to elucidate the mechanisms of cardiac damage due to COVID-19.

This study has limitations. First, in the absence of a COVID-19–negative control group, it was not possible to quantify an increased risk in CVEs precisely due to COVID-19. Second, these results cannot be generalized to reflect the risk of CVE exacerbation for patients with a preexisting cardiovascular condition or patients who died within 30 days of COVID-19 diagnosis, as these individuals were excluded from our analyses. Furthermore, exclusion of these individuals, and those with CVEs in the prior year, may not fully prevent carryover and may underestimate the number of CVEs, potentially biasing our results toward the null. Despite this limitation, these methods have been used in prior studies to report the risk of cardiovascular-associated mortality after bacterial pneumonia [45]. Third, it is possible that residual or unmeasured confounding remains beyond the balance we were able to demonstrate with the stabilized propensity scores. Specifically, as is common in claims-based analyses in the United States, COVID-19 vaccination status was underreported compared to publicly available vaccine uptake estimates. Thus, we were unable to account for vaccination in our analyses beyond including an indicator in the propensity score for whether or not a patient had received at least 1 COVID vaccine dose based on administrative claims. Additionally, it is possible that confounding by COVID-19 treatment status may have biased our results, particularly in the outpatient setting. However, COVID-19 treatment was rare in our study (<5% for dexamethasone; <1% each for monoclonal antibodies and remdesivir; Supplementary Table 17), and likely had minimal impact on the results. Our use of a composite endpoint encompassing a broad range of CVEs may make it difficult to tease apart the distinct events or organ impacts of COVID-19. Last, the HealthVerity dataset has limited capture of in-hospital deaths and does not capture out-of-hospital deaths.

In conclusion, we found that patients diagnosed with COVID-19 who were hospitalized or required ICU care had a significantly higher risk of experiencing and being hospitalized for post–COVID-19 cardiac events than those treated in the outpatient setting only. This finding showed dose-response and persisted after controlling for demographic and clinical characteristics differences. These findings underscore the continued importance of preventing SARS-CoV-2 infection from progressing to severe illness to reduce potential long-term cardiovascular complications.

## Supplementary Data

Supplementary materials are available at *Clinical Infectious Diseases* online. Consisting of data provided by the authors to benefit the reader, the posted materials are not copyedited and are the sole responsibility of the authors, so questions or comments should be addressed to the corresponding author.

## Supplementary Material

ciac661_Supplementary_Data

## Notes

***Author Contributions*.** T. L. W., J. M. L., L. J. M., K. M. A., and F. K were responsible for study concept and design. All authors contributed to acquisition, analysis, or interpretation of data. T. L. W., L. J. G., K. M. A., and J. M. L. wrote the first draft. All authors contributed to drafting of the manuscript and critical revision of the manuscript for important intellectual content. All authors provided administrative, technical, or material support. J. M. L. provided supervision. T. L. W., L. J. M., and F. K. had full access to all the data in the study and take responsibility for the data's integrity and the accuracy of the data analysis.

***Acknowledgments*.** The authors thank Maya Reimbaeva of Pfizer Inc, Ziphora Sam of Eliassen Group (Somerset, New Jersey), and Darren Jeng of Pfizer Inc for their contributions to conducting the statistical programming for this project.

***Financial support.*** This work was supported by Pfizer Inc.
