# Supplementary material for: Coronavirus Disease 2019 Severity and Risk of Subsequent Cardiovascular Events
Source: Clin Infect Dis. 2022 Sep 5;76(3):e42–50. doi: 10.1093/cid/ciac661 (PMC9907540; doi:10.1093/cid/ciac661)
Supplement: ciac661_Supplementary_Data [file ciac661_supplementary_data.docx]

Supplementary Appendix

[Table S1. ICD-10-CM codes used to identify outcomes 2](#_Toc109229360)

[Table S2. Baseline characteristics of patients diagnosed with COVID-19 aged 18-49 4](#_Toc109229361)

[Table S3. Baseline characteristics of patients diagnosed with COVID-19 aged 50-64 6](#_Toc109229362)

[Table S4. Baseline characteristics of patients diagnosed with COVID-19 aged 65+ 8](#_Toc109229363)

[Table S5. Distribution of stabilized weights for primary analysis, 18+ 10](#_Toc109229364)

[Table S6. Distribution of follow-up visits 11](#_Toc109229365)

[Table S7. Risk of cardiovascular events by COVID-19 level of care setting, 18+ 12](#_Toc109229366)

[Table S8. Risk of cardiovascular events requiring inpatient care by COVID-19 level of care setting, 18+ 13](#_Toc109229367)

[Table S9. E-values for strength of association between acute disease severity and risk of cardiac complications 14](#_Toc109229368)

[Table S10. Risk of alternative categorization of cardiovascular events by COVID-19 level of care setting, 18+ 15](#_Toc109229369)

[Table S11. Risk of cardiovascular events by COVID-19 level of care setting, 18-49 16](#_Toc109229370)

[Table S12. Risk of cardiovascular events by COVID-19 level of care setting, 50-64 17](#_Toc109229371)

[Table S13. Risk of cardiovascular events by COVID-19 level of care setting, 65+ 18](#_Toc109229372)

[Table S14. Risk of cardiovascular events requiring inpatient care by COVID-19 level of care setting, 18-49 19](#_Toc109229373)

[Table S15. Risk of cardiovascular events requiring inpatient care by COVID-19 level of care setting, 50-64 20](#_Toc109229374)

[Table S16. Risk of cardiovascular events requiring inpatient care by COVID-19 level of care setting, 65+ 21](#_Toc109229375)

[Figure S1. Risk of individual cardiovascular events by COVID-19 level of care setting, 18+ 22](#_Toc109229376)

[Table S17. Number of patients with COVID treatment and specific outcome codes 23](#_Toc109229377)

Table S1. ICD-10-CM codes used to identify outcomes

| **Cardiovascular Event** | **ICD-10-CM Codes** | **Description** |
| --- | --- | --- |
| Cerebrovascular accident: ischemic stroke, hemorrhagic stroke, TIA | I60.xx | Nontraumatic subarachnoid hemorrhage |
|  | I61.xx | Nontraumatic intracerebral hemorrhage |
|  | I62.xx | Other and unspecified nontraumatic intracranial hemorrhage |
|  | I63.xx | Cerebral infarction |
|  | I65.xx | Occlusion and stenosis of precerebral arteries, not resulting in cerebral infarction |
|  | I66.xx | Occlusion and stenosis of cerebral arteries, not resulting in cerebral infarction |
|  | I67.0 | Dissection of cerebral arteries, nonruptured |
|  | I67.4 | Hypertensive encephalopathy |
|  | I67.6 | Nonpyogenic thrombosis of intracranial venous system |
|  | I67.81 | Acute cerebrovascular insufficiency |
|  | I67.82 | Cerebral ischemia |
|  | I67.841 | Reversible cerebrovascular vasoconstriction syndrome |
|  | G45.8 | Other transient cerebral ischemic attacks and related syndromes |
|  | G45.9 | Transient cerebral ischemic attack, unspecified |
| Dysrhythmia | I47.xx | Paroxysmal tachycardia |
|  | I48.xx | Atrial fibrillation and flutter |
|  | I49.xx | Other cardiac arrhythmias |
|  | R00.xx | Abnormalities of heart beat |
| Ischemic heart disease: acute coronary disease, myocardial infarction, ischemic cardiomyopathy, angina | I20.xx | Angina pectoris |
|  | I21.xx | Acute myocardial infarction |
|  | I22.xx | Subsequent ST elevation (STEMI) and non-ST elevation (NSTEMI) myocardial infarction |
|  | I23.xx | Certain current complications following ST elevation (STEMI) and non-ST elevation (NSTEMI) myocardial infarction (within the 28 day period) |
|  | I24.xx | Other acute ischemic heart diseases |
|  | I25.xx, excluding I25.2 and I25.9 | Chronic ischemic heart disease |
| Other: myocarditis/pericarditis, heart failure, cardiomyopathy, cardiac arrest, cardiogenic shock | B33.2x | Viral carditis |
|  | I40.xx | Acute myocarditis |
|  | I41 | Myocarditis in diseases classified elsewhere |
|  | I51.4 | Myocarditis, unspecified |
|  | I30.1 | Infective pericarditis |
|  | I09.81 | Rheumatic heart failure |
|  | I11.0 | Hypertensive heart disease with heart failure |
|  | I13.0 | Hypertensive heart and chronic kidney disease with heart failure and stage 1 through stage 4 chronic kidney disease, or unspecified chronic kidney disease |
|  | I13.2 | Hypertensive heart and chronic kidney disease with heart failure and with stage 5 chronic kidney disease, or end stage renal disease |
|  | I50.xx | Heart failure |
|  | I42.xx | Cardiomyopathy |
|  | I43 | Cardiomyopathy in diseases classified elsewhere |
|  | I46.xx | Cardiac arrest |
|  | R57.0 | Cardiogenic shock |
| Thrombotic disorders: pulmonary embolism, deep vein thrombosis, superficial vein thrombosis | D65 | Disseminated intravascular coagulation [defibrination syndrome] |
|  | I26.xx | Pulmonary embolism |
|  | I74.xx | Arterial embolism and thrombosis |
|  | I75.xx | Atheroembolism |
|  | I80.xx | Phlebitis and thrombophlebitis |
|  | I82.xx | Other venous embolism and thrombosis |
| ICD-10-CM, International Classification of Diseases, 10th edition, clinical modification; TIA, Transient ischemic attack; x and xx indicate all subcodes. | | |

# Table S2. Baseline characteristics of patients diagnosed with COVID-19 aged 18-49

|  | **Outpatient**  **(n=876,333)** | **Inpatient/ICU-**  **(n=20,964)** | **Inpatient/ICU+**  **(n=7,759)** | **Weighted SMD:**  **Inpatient/ICU-**  **vs Outpatient** | **Weighted SMD:**  **Inpatient/ICU+**  **vs Outpatient** |
| --- | --- | --- | --- | --- | --- |
| Mean Age, years (Std) | 32.5 (9.4) | 34.1 (9.0) | 37.2 (8.7) | 0.108 | 0.133 |
| Sex |  |  |  |  |  |
| Female | 537,879 (61.4) | 14,058 (67.1) | 4,121 (53.1) | 0.068 | 0.027 |
| Male | 334,032 (38.1) | 6,698 (31.9) | 3,588 (46.2) |  |  |
| Unknown | 4,422 (0.5) | 208 (1.0) | 50 (0.6) |  |  |
| Region |  |  |  |  |  |
| Northeast | 162,182 (18.5) | 4,158 (19.8) | 1,346 (17.3) | 0.012 | 0.047 |
| Midwest | 203,912 (23.3) | 4,707 (22.5) | 1,543 (19.9) |  |  |
| South | 403,977 (46.1) | 9,474 (45.2) | 3,766 (48.5) |  |  |
| West | 106,005 (12.1) | 2,619 (12.5) | 1,099 (14.2) |  |  |
| Other | 193 (<0.1) | 4 (<0.1) | 4 (<0.1) |  |  |
| Missing | 64 (<0.1) | 2 (<0.1) | 1 (<0.1) |  |  |
| 1+ COVID+ vaccine dose | 7,480 (0.9) | 123 (0.6) | 47 (0.6) | -0.042 | -0.049 |
| Indicators of poor health |  |  |  |  |  |
| Smoking | 75,407 (8.6) | 3,051 (14.6) | 960 (12.4) | 0.022 | 0.035 |
| Obesity | 162,479 (18.5) | 6,510 (31.1) | 2,649 (34.1) | 0.071 | 0.101 |
| Nursing home residence | 1,363 (0.2) | 83 (0.4) | 41 (0.5) | 0.019 | 0.005 |
| Skilled nursing facility | 1,706 (0.2) | 117 (0.6) | 44 (0.6) | 0.004 | 0.008 |
| Prior hospitalization | 60,301 (6.9) | 4,110 (19.6) | 1,369 (17.6) | 0.020 | 0.004 |
| Impaired functional status | 8,317 (0.9) | 554 (2.6) | 295 (3.8) | 0.017 | 0.036 |
| Indicator of health-seeking behavior |  |  |  |  |  |
| Pneumococcal vaccine | 6,427 (0.7) | 272 (1.3) | 139 (1.8) | 0.004 | 0.017 |
| Influenza vaccine | 250,927 (28.6) | 5,829 (27.8) | 2,079 (26.8) | 0.024 | 0.009 |
| Wellness visit | 529,786 (60.5) | 11,685 (55.7) | 4,120 (53.1) | 0.018 | -0.002 |
| Hypertension | 134,261 (15.3) | 5,009 (23.9) | 2,638 (34.0) | 0.040 | 0.058 |
| Comorbid conditions |  |  |  |  |  |
| Myocardial infarction | 1,043 (0.1) | 64 (0.3) | 34 (0.4) | 0.003 | 0.002 |
| Congestive heart failure | 1,565 (0.2) | 98 (0.5) | 64 (0.8) | -0.001 | 0.001 |
| Peripheral vascular disease | 6,337 (0.7) | 355 (1.7) | 177 (2.3) | 0.006 | 0.011 |
| Cerebrovascular disease | 4,378 (0.5) | 172 (0.8) | 102 (1.3) | 0.007 | 0.025 |
| Dementia | 709 (0.1) | 54 (0.3) | 23 (0.3) | 0.002 | 0.020 |
| Chronic pulmonary disease | 121,870 (13.9) | 3,844 (18.3) | 1,552 (20.0) | 0.025 | 0.052 |
| Rheumatic disease | 11,883 (1.4) | 437 (2.1) | 196 (2.5) | 0.016 | 0.020 |
| Peptic ulcer disease | 5,231 (0.6) | 211 (1.0) | 88 (1.1) | 0.009 | 0.005 |
| Mild liver disease | 38,611 (4.4) | 1,607 (7.7) | 797 (10.3) | 0.030 | 0.053 |
| Diabetes without complications | 60,147 (6.9) | 3,026 (14.4) | 1,851 (23.9) | 0.039 | 0.072 |
| Diabetes with complications | 12,766 (1.5) | 915 (4.4) | 626 (8.1) | 0.017 | 0.030 |
| Hemiplegia or paraplegia | 2,474 (0.3) | 172 (0.8) | 104 (1.3) | 0.009 | 0.024 |
| Any malignancy | 10,143 (1.2) | 397 (1.9) | 172 (2.2) | 0.009 | 0.019 |
| Moderate/severe liver disease | 689 (0.1) | 73 (0.3) | 57 (0.7) | 0.003 | 0.006 |
| Metastatic solid tumor | 1,433 (0.2) | 84 (0.4) | 35 (0.5) | 0.004 | 0.009 |
| HIV/AIDS | 3,920 (0.4) | 197 (0.9) | 78 (1.0) | 0.002 | 0.008 |
| CKD/ESRD | 25,676 (2.9) | 1,398 (6.7) | 729 (9.4) | 0.017 | 0.029 |
| High-risk immunocompromised conditions |  |  |  |  |  |
| Solid malignancy | 75,988 (8.7) | 1,744 (8.3) | 713 (9.2) | 0.020 | 0.030 |
| Hematologic malignancy | 1,099 (0.1) | 61 (0.3) | 28 (0.4) | 0.001 | 0.010 |
| Bone marrow transplant | 96 (<0.1) | 3 (<0.1) | 7 (0.1) | -0.002 | -0.006 |
| Organ transplant | 1,203 (0.1) | 152 (0.7) | 89 (1.1) | 0.006 | 0.012 |
| Rheumatologic or other inflammatory condition | 56,550 (6.5) | 1,729 (8.2) | 752 (9.7) | 0.018 | 0.045 |
| Primary immunodeficiency | 12,087 (1.4) | 710 (3.4) | 362 (4.7) | 0.009 | 0.019 |
| Other immune condition | 17,403 (2.0) | 849 (4.0) | 319 (4.1) | 0.003 | 0.009 |
| Immunosuppressive medication >= 14 days | 42,557 (4.9) | 1,228 (5.9) | 627 (8.1) | 0.021 | 0.017 |
| Antimetabolite medication >= 14 days | 4,013 (0.5) | 211 (1.0) | 111 (1.4) | 0.009 | 0.011 |

Continuous variables are shown as mean (standard deviation), and categorical variables as counts (percentages). Indicators of poor health, hypertension (diagnosis code and/or anti-hypertensive medication) and high-risk immunocompromised conditions were measured in the year prior to COVID diagnosis. Indicators of health-seeking behavior and comorbid conditions were assessed using all-available lookback data. SMD: standardized mean difference, where >0.10 indicates a significant difference.

# Table S3. Baseline characteristics of patients diagnosed with COVID-19 aged 50-64

|  | **Outpatient**  **(n=329,024)** | **Inpatient/ICU-**  **(n=15,067)** | **Inpatient/ICU+**  **(n=8,834)** | **Weighted SMD:**  **Inpatient/ICU-**  **vs Outpatient** | **Weighted SMD:**  **Inpatient/ICU+**  **vs Outpatient** |
| --- | --- | --- | --- | --- | --- |
| Mean Age, years (Std) | 56.5 (4.2) | 57.2 (4.2) | 57.3 (4.2) | 0.027 | 0.048 |
| Sex |  |  |  | 0.019 | 0.033 |
| Female | 189,755 (57.7) | 7,753 (51.5) | 4,338 (49.1) |  |  |
| Male | 138,209 (42.0) | 7,222 (47.9) | 4,460 (50.5) |  |  |
| Unknown | 1,060 (0.3) | 92 (0.6) | 36 (0.4) |  |  |
| Region |  |  |  | 0.025 | 0.046 |
| Northeast | 68,486 (20.8) | 3,428 (22.8) | 1,635 (18.5) |  |  |
| Midwest | 76,500 (23.3) | 3,471 (23.0) | 1,836 (20.8) |  |  |
| South | 144,927 (44.0) | 6,441 (42.7) | 4,090 (46.3) |  |  |
| West | 38,592 (11.7) | 1,709 (11.3) | 1,271 (14.4) |  |  |
| Other | 499 (0.2) | 17 (0.1) | 2 ((<0.1) |  |  |
| Missing | 20 (<0.1) | 1 ((<0.1) | 0 (0.0) |  |  |
| 1+ COVID+ vaccine dose | 4,965 (1.5) | 163 (1.1) | 90 (1.0) | -0.058 | -0.049 |
| Indicators of poor health |  |  |  |  |  |
| Smoking | 33,469 (10.2) | 2,162 (14.3) | 1,211 (13.7) | 0.017 | 0.009 |
| Obesity | 82,590 (25.1) | 4,920 (32.7) | 3,116 (35.3) | 0.044 | 0.053 |
| Nursing home residence | 3,299 (1.0) | 229 (1.5) | 146 (1.7) | 0.010 | 0.019 |
| Skilled nursing facility | 3,862 (1.2) | 254 (1.7) | 165 (1.9) | 0.010 | 0.023 |
| Prior hospitalization | 16,487 (5.0) | 2,280 (15.1) | 1,215 (13.8) | 0.007 | -0.009 |
| Impaired functional status | 10,405 (3.2) | 999 (6.6) | 577 (6.5) | 0.019 | 0.020 |
| Indicator of health-seeking behavior |  |  |  |  |  |
| Pneumococcal vaccine | 10,904 (3.3) | 614 (4.1) | 414 (4.7) | 0.013 | 0.016 |
| Influenza vaccine | 140,160 (42.6) | 5,846 (38.8) | 3,469 (39.3) | 0.008 | 0.005 |
| Wellness visit | 219,966 (66.9) | 8,748 (58.1) | 5,112 (57.9) | -0.015 | -0.022 |
| Hypertension | 156,955 (47.7) | 8,745 (58.0) | 5,392 (61.0) | 0.043 | 0.040 |
| Comorbid conditions |  |  |  |  |  |
| Myocardial infarction | 1,600 (0.5) | 87 (0.6) | 60 (0.7) | 0.011 | 0.000 |
| Congestive heart failure | 2,162 (0.7) | 204 (1.4) | 108 (1.2) | 0.001 | 0.007 |
| Peripheral vascular disease | 14,325 (4.4) | 1,048 (7.0) | 641 (7.3) | 0.015 | -0.003 |
| Cerebrovascular disease | 6,803 (2.1) | 458 (3.0) | 250 (2.8) | 0.007 | -0.006 |
| Dementia | 2,304 (0.7) | 188 (1.2) | 132 (1.5) | 0.002 | 0.006 |
| Chronic pulmonary disease | 55,950 (17.0) | 3,479 (23.1) | 2,036 (23.0) | 0.031 | 0.025 |
| Rheumatic disease | 11,976 (3.6) | 650 (4.3) | 399 (4.5) | 0.014 | -0.002 |
| Peptic ulcer disease | 4,202 (1.3) | 312 (2.1) | 166 (1.9) | 0.011 | 0.019 |
| Mild liver disease | 30,826 (9.4) | 1,941 (12.9) | 1,239 (14.0) | 0.022 | 0.034 |
| Diabetes without complications | 75,566 (23.0) | 5,208 (34.6) | 3,455 (39.1) | 0.036 | 0.041 |
| Diabetes with complications | 25,582 (7.8) | 2,170 (14.4) | 1,557 (17.6) | 0.021 | 0.026 |
| Hemiplegia or paraplegia | 1,708 (0.5) | 163 (1.1) | 125 (1.4) | 0.005 | 0.006 |
| Any malignancy | 17,307 (5.3) | 954 (6.3) | 539 (6.1) | 0.016 | 0.015 |
| Moderate/severe liver disease | 1,345 (0.4) | 176 (1.2) | 109 (1.2) | 0.010 | 0.011 |
| Metastatic solid tumor | 2,367 (0.7) | 199 (1.3) | 106 (1.2) | 0.004 | 0.016 |
| HIV/AIDS | 2,702 (0.8) | 221 (1.5) | 105 (1.2) | 0.000 | -0.008 |
| CKD/ESRD | 26,377 (8.0) | 2,115 (14.0) | 1,351 (15.3) | 0.022 | 0.014 |
| High-risk immunocompromised conditions |  |  |  |  |  |
| Solid malignancy | 69,978 (21.3) | 2,793 (18.5) | 1,601 (18.1) | -0.004 | -0.002 |
| Hematologic malignancy | 1,419 (0.4) | 139 (0.9) | 73 (0.8) | 0.006 | 0.013 |
| Bone marrow transplant | 70 (0.0) | 11 (0.1) | 2 (-<0.1) | 0.008 | 0.005 |
| Organ transplant | 1,000 (0.3) | 173 (1.1) | 81 (0.9) | 0.004 | 0.003 |
| Rheumatologic or other inflammatory condition | 37,042 (11.3) | 2,098 (13.9) | 1,225 (13.9) | 0.020 | 0.010 |
| Primary immunodeficiency | 6,053 (1.8) | 535 (3.6) | 325 (3.7) | -0.001 | -0.012 |
| Other immune condition | 10,289 (3.1) | 623 (4.1) | 362 (4.1) | -0.004 | -0.021 |
| Immunosuppressive medication >= 14 days | 27,951 (8.5) | 1,637 (10.9) | 973 (11.0) | 0.030 | 0.010 |
| Antimetabolite medication >= 14 days | 4,022 (1.2) | 276 (1.8) | 160 (1.8) | 0.016 | 0.003 |

Continuous variables are shown as mean (standard deviation), and categorical variables as counts (percentages). Indicators of poor health, hypertension (diagnosis code and/or anti-hypertensive medication) and high-risk immunocompromised conditions were measured in the year prior to COVID diagnosis. Indicators of health-seeking behavior and comorbid conditions were assessed using all-available lookback data. SMD: standardized mean difference, where >0.10 indicates a significant difference.

# Table S4. Baseline characteristics of patients diagnosed with COVID-19 aged 65+

|  | **Outpatient**  **(n=86,707)** | **Inpatient/ICU-**  **(n=8,354)** | **Inpatient/ICU+**  **(n=4,476)** | **Weighted SMD:**  **Inpatient/ICU-**  **vs Outpatient** | **Weighted SMD:**  **Inpatient/ICU+**  **vs Outpatient** |
| --- | --- | --- | --- | --- | --- |
| Mean Age, years (Std) | 73.4 (8.5) | 75.2 (9.0) | 74.3 (8.6) | 0.055 | 0.049 |
| Sex |  |  |  | 0.007 | 0.004 |
| Female | 53,711 (61.9) | 4,982 (59.6) | 2,452 (54.8) |  |  |
| Male | 32,758 (37.8) | 3,338 (40.0) | 2,002 (44.7) |  |  |
| Unknown | 238 (0.3) | 34 (0.4) | 22 (0.5) |  |  |
| Region |  |  |  | 0.042 | 0.033 |
| Northeast | 22,514 (26.0) | 2,502 (29.9) | 987 (22.1) |  |  |
| Midwest | 15,770 (18.2) | 1,445 (17.3) | 751 (16.8) |  |  |
| South | 35,051 (40.4) | 3,442 (41.2) | 2,017 (45.1) |  |  |
| West | 10,925 (12.6) | 867 (10.4) | 701 (15.7) |  |  |
| Other | 2,436 (2.8) | 98 (1.2) | 18 (0.4) |  |  |
| Missing | 11 (<0.1) | 0 (0.0) | 2 (<0.1) |  |  |
| 1+ COVID+ vaccine dose | 1,088 (1.3) | 64 (0.8) | 34 (0.8) | -0.050 | -0.028 |
| Indicators of poor health |  |  |  |  |  |
| Smoking | 9,413 (10.9) | 1,156 (13.8) | 618 (13.8) | 0.004 | 0.006 |
| Obesity | 18,948 (21.9) | 1,974 (23.6) | 1,268 (28.3) | 0.006 | 0.008 |
| Nursing home residence | 9,284 (10.7) | 597 (7.1) | 279 (6.2) | 0.041 | 0.035 |
| Skilled nursing facility | 11,667 (13.5) | 768 (9.2) | 328 (7.3) | 0.039 | 0.030 |
| Prior hospitalization | 8,054 (9.3) | 1,559 (18.7) | 761 (17.0) | 0.020 | 0.017 |
| Impaired functional status | 17,387 (20.1) | 1,862 (22.3) | 825 (18.4) | 0.037 | 0.047 |
| Indicator of health-seeking behavior |  |  |  |  |  |
| Pneumococcal vaccine | 15,383 (17.7) | 1,282 (15.3) | 746 (16.7) | -0.002 | -0.020 |
| Influenza vaccine | 42,195 (48.7) | 3,623 (43.4) | 2,008 (44.9) | -0.008 | -0.022 |
| Wellness visit | 54,331 (62.7) | 4,655 (55.7) | 2,652 (59.2) | -0.005 | -0.014 |
| Hypertension | 61,268 (70.8) | 6,180 (74.0) | 3,381 (75.5) | 0.029 | 0.035 |
| Comorbid conditions |  |  |  |  |  |
| Myocardial infarction | 845 (1.0) | 98 (1.2) | 39 (0.9) | 0.002 | -0.004 |
| Congestive heart failure | 1,862 (2.1) | 209 (2.5) | 115 (2.6) | 0.003 | 0.013 |
| Peripheral vascular disease | 19,407 (22.4) | 1,875 (22.4) | 1,121 (25.0) | 0.028 | 0.020 |
| Cerebrovascular disease | 6,188 (7.1) | 660 (7.9) | 314 (7.0) | 0.015 | 0.027 |
| Dementia | 12,428 (14.3) | 1,310 (15.7) | 579 (12.9) | 0.022 | 0.011 |
| Chronic pulmonary disease | 19,497 (22.5) | 2,163 (25.9) | 1,190 (26.6) | 0.028 | 0.031 |
| Rheumatic disease | 4,680 (5.4) | 415 (5.0) | 212 (4.7) | 0.006 | 0.020 |
| Peptic ulcer disease | 1,654 (1.9) | 206 (2.5) | 99 (2.2) | 0.005 | 0.006 |
| Mild liver disease | 7,998 (9.2) | 861 (10.3) | 496 (11.1) | 0.014 | -0.008 |
| Diabetes without complications | 32,502 (37.5) | 3,622 (43.4) | 2,067 (46.2) | 0.019 | 0.011 |
| Diabetes with complications | 16,632 (19.2) | 2,071 (24.8) | 1,265 (28.3) | 0.021 | 0.011 |
| Hemiplegia or paraplegia | 1,122 (1.3) | 157 (1.9) | 71 (1.6) | 0.001 | 0.022 |
| Any malignancy | 8,815 (10.2) | 1,001 (12.0) | 544 (12.2) | 0.006 | -0.008 |
| Moderate/severe liver disease | 533 (0.6) | 72 (0.9) | 45 (1.0) | 0.009 | -0.005 |
| Metastatic solid tumor | 976 (1.1) | 148 (1.8) | 79 (1.8) | 0.002 | -0.008 |
| HIV/AIDS | 394 (0.5) | 52 (0.6) | 27 (0.6) | -0.003 | -0.001 |
| CKD/ESRD | 18,518 (21.4) | 2,385 (28.5) | 1,367 (30.5) | 0.007 | 0.025 |
| High-risk immunocompromised conditions |  |  |  |  |  |
| Solid malignancy | 21,053 (24.3) | 1,989 (23.8) | 1,027 (22.9) | 0.004 | -0.013 |
| Hematologic malignancy | 709 (0.8) | 113 (1.4) | 83 (1.9) | -0.002 | 0.000 |
| Bone marrow transplant | 26 (<0.1) | 5 (0.1) | 3 (0.1) | 0.000 | -0.011 |
| Organ transplant | 330 (0.4) | 74 (0.9) | 32 (0.7) | 0.001 | -0.006 |
| Rheumatologic or other inflammatory condition | 12,318 (14.2) | 1,238 (14.8) | 714 (16.0) | 0.015 | 0.017 |
| Primary immunodeficiency | 2,340 (2.7) | 344 (4.1) | 222 (5.0) | 0.014 | 0.000 |
| Other immune condition | 2,758 (3.2) | 351 (4.2) | 224 (5.0) | 0.010 | -0.013 |
| Immunosuppressive medication >= 14 days | 7,804 (9.0) | 751 (9.0) | 467 (10.4) | 0.013 | 0.007 |
| Antimetabolite medication >= 14 days | 917 (1.1) | 112 (1.3) | 59 (1.3) | 0.000 | 0.008 |

Continuous variables are shown as mean (standard deviation), and categorical variables as counts (percentages). Indicators of poor health, hypertension (diagnosis code and/or anti-hypertensive medication) and high-risk immunocompromised conditions were measured in the year prior to COVID diagnosis. Indicators of health-seeking behavior and comorbid conditions were assessed using all-available lookback data. SMD: standardized mean difference, where >0.10 indicates a significant difference.

# Table S5. Distribution of stabilized weights for primary analysis, 18+

|  | **Outpatient** | **Inpatient/ICU-** | **Inpatient/ICU+** |
| --- | --- | --- | --- |
| Mean (Std) | 1.00 (0.06) | 0.97 (0.71) | 0.94 (1.03) |
| Minimum/Maximum | 0.95, 6.14 | 0.05, 5.52 | 0.04, 16.17 |
| First Quartile/Third Quartile | 0.97, 1.01 | 0.43, 1.33 | 0.32, 1.14 |
| Median (IQR) | 0.98 (0.03) | 0.80 (0.90) | 0.61 (0.82) |

# Table S6. Distribution of follow-up visits

|  | **Total** | **Mild** | **Moderate** | **Severe** |
| --- | --- | --- | --- | --- |
| Total number of patients | **1,357,518** | **1,292,064** | **44,385** | **21,069** |
| Inpatient visits in first 6 months of follow-up | | |  |  |
| Median | 1 | 1 | 1 | 1 |
| Interquartile range | 1-2 | 1-2 | 1-2 | 1-2 |
| Outpatient visits in first 6 months of follow-up | | |  |  |
| Median | 5 | 5 | 7 | 9 |
| Interquartile range | 2-10 | 2-10 | 3-16 | 4-19 |

There were 1,289,542 persons who did not have any inpatient (1,233,477 in the mild group, 38,522 moderate and 17,543 severe) and 259,239 persons who did not have any outpatient (248,529 mild, 7,779 moderate and 2,931 severe) visits in the 6 months after the 30-day assessment period. These persons are not considered in the table above.

# Table S7. Risk of cardiovascular events by COVID-19 level of care setting, 18+

|  | | | | **Weighted 9-month incidence (95% CI)** | | | **Unweighted Hazard Ratio (95% CI)** | | | **Weighted Hazard Ratio (95% CI)** | | |
| --- | --- | --- | --- | --- | --- | --- | --- | --- | --- | --- | --- | --- |
| **Endpoint** | **COVID status** | **Number of events** | **Number of deaths** | **Incidence** | **LCL** | **UCL** | **Hazard Ratio** | **LCL** | **UCL** | **Hazard Ratio** | **LCL** | **UCL** |
| Composite CV Event | Outpatient | 107728 | 43 | 0.075 | 0.075 | 0.075 | - | - | - | - | - | - |
|  | Inpatient/ICU- | 6557 | 8 | 0.097 | 0.094 | 0.100 | 1.85 | 1.80 | 1.90 | 1.28 | 1.24 | 1.33 |
|  | Inpatient/ICU+ | 4065 | 13 | 0.138 | 0.131 | 0.144 | 2.49 | 2.41 | 2.57 | 1.80 | 1.71 | 1.89 |
| Dysrhythmia | Outpatient | 73887 | 107 | 0.051 | 0.050 | 0.051 | - | - | - | - | - | - |
|  | Inpatient/ICU- | 3899 | 19 | 0.063 | 0.060 | 0.066 | 1.57 | 1.52 | 1.62 | 1.22 | 1.17 | 1.27 |
|  | Inpatient/ICU+ | 2458 | 26 | 0.092 | 0.087 | 0.098 | 2.11 | 2.02 | 2.19 | 1.75 | 1.65 | 1.87 |
| Ischemic heart disease | Outpatient | 19839 | 155 | 0.014 | 0.014 | 0.014 | - | - | - | - | - | - |
|  | Inpatient/ICU- | 1490 | 29 | 0.018 | 0.016 | 0.019 | 2.20 | 2.09 | 2.32 | 1.24 | 1.16 | 1.32 |
|  | Inpatient/ICU+ | 984 | 33 | 0.023 | 0.021 | 0.026 | 3.05 | 2.86 | 3.26 | 1.59 | 1.45 | 1.74 |
| Other cardiac disorders and myocarditis/pericarditis | Outpatient | 10716 | 86 | 0.008 | 0.007 | 0.008 | - | - | - | - | - | - |
|  | Inpatient/ICU- | 1225 | 21 | 0.013 | 0.012 | 0.014 | 3.35 | 3.16 | 3.56 | 1.64 | 1.52 | 1.77 |
|  | Inpatient/ICU+ | 915 | 21 | 0.021 | 0.019 | 0.023 | 5.27 | 4.92 | 5.64 | 2.66 | 2.42 | 2.94 |
| Thrombotic disorders | Outpatient | 7955 | 170 | 0.005 | 0.005 | 0.006 | - | - | - | - | - | - |
|  | Inpatient/ICU- | 752 | 32 | 0.010 | 0.009 | 0.011 | 2.76 | 2.56 | 2.97 | 1.76 | 1.60 | 1.94 |
|  | Inpatient/ICU+ | 506 | 40 | 0.015 | 0.013 | 0.017 | 3.88 | 3.55 | 4.25 | 2.51 | 2.21 | 2.84 |
| Cerebrovascular accident | Outpatient | 21422 | 160 | 0.015 | 0.014 | 0.015 | - | - | - | - | - | - |
|  | Inpatient/ICU- | 1672 | 30 | 0.021 | 0.019 | 0.022 | 2.29 | 2.18 | 2.41 | 1.36 | 1.27 | 1.45 |
|  | Inpatient/ICU+ | 1113 | 38 | 0.029 | 0.027 | 0.032 | 3.21 | 3.02 | 3.41 | 1.90 | 1.74 | 2.08 |

# Table S8. Risk of cardiovascular events requiring inpatient care by COVID-19 level of care setting, 18+

|  | | | | **Weighted 9-month incidence (95% CI)** | | | **Unweighted Hazard Ratio (95% CI)** | | | **Weighted Hazard Ratio (95% CI)** | | |
| --- | --- | --- | --- | --- | --- | --- | --- | --- | --- | --- | --- | --- |
| **Endpoint** | **COVID status** | **Number of events** | **Number of deaths** | **Incidence** | **LCL** | **UCL** | **Hazard Ratio** | **LCL** | **UCL** | **Hazard Ratio** | **LCL** | **UCL** |
| Composite CV Event | Outpatient | 16122 | 43 | 0.012 | 0.012 | 0.012 | - | - | - | - | - | - |
|  | Inpatient/ICU- | 1896 | 8 | 0.024 | 0.023 | 0.026 | 3.57 | 3.41 | 3.75 | 1.96 | 1.85 | 2.09 |
|  | Inpatient/ICU+ | 1375 | 13 | 0.043 | 0.039 | 0.047 | 5.62 | 5.32 | 5.95 | 3.47 | 3.20 | 3.76 |
| Dysrhythmia | Outpatient | 9841 | 107 | 0.007 | 0.007 | 0.007 | - | - | - | - | - | - |
|  | Inpatient/ICU- | 1142 | 19 | 0.015 | 0.014 | 0.016 | 3.44 | 3.24 | 3.66 | 2.02 | 1.86 | 2.18 |
|  | Inpatient/ICU+ | 844 | 26 | 0.028 | 0.025 | 0.031 | 5.43 | 5.06 | 5.83 | 3.68 | 3.32 | 4.08 |
| Ischemic heart disease | Outpatient | 4401 | 155 | 0.003 | 0.003 | 0.003 | - | - | - | - | - | - |
|  | Inpatient/ICU- | 496 | 29 | 0.005 | 0.004 | 0.006 | 3.30 | 3.01 | 3.62 | 1.57 | 1.40 | 1.77 |
|  | Inpatient/ICU+ | 350 | 33 | 0.007 | 0.006 | 0.009 | 4.88 | 4.38 | 5.45 | 2.30 | 1.96 | 2.69 |
| Other cardiac disorders and myocarditis/pericarditis | Outpatient | 3485 | 86 | 0.003 | 0.002 | 0.003 | - | - | - | - | - | - |
|  | Inpatient/ICU- | 525 | 21 | 0.005 | 0.004 | 0.006 | 4.42 | 4.03 | 4.84 | 1.92 | 1.71 | 2.16 |
|  | Inpatient/ICU+ | 455 | 21 | 0.010 | 0.009 | 0.012 | 8.05 | 7.30 | 8.88 | 3.95 | 3.45 | 4.51 |
| Thrombotic disorders | Outpatient | 2416 | 170 | 0.002 | 0.002 | 0.002 | - | - | - | - | - | - |
|  | Inpatient/ICU- | 345 | 32 | 0.004 | 0.003 | 0.004 | 4.15 | 3.71 | 4.65 | 2.04 | 1.77 | 2.35 |
|  | Inpatient/ICU+ | 252 | 40 | 0.007 | 0.006 | 0.008 | 6.35 | 5.58 | 7.24 | 3.89 | 3.27 | 4.63 |
| Cerebrovascular accident | Outpatient | 5980 | 160 | 0.004 | 0.004 | 0.004 | - | - | - | - | - | - |
|  | Inpatient/ICU- | 627 | 30 | 0.007 | 0.006 | 0.008 | 3.08 | 2.84 | 3.35 | 1.64 | 1.48 | 1.82 |
|  | Inpatient/ICU+ | 483 | 38 | 0.012 | 0.011 | 0.014 | 5.01 | 4.56 | 5.49 | 2.75 | 2.41 | 3.13 |

Table S9. E-values for strength of association between acute disease severity and risk of cardiac complications

| **Endpoint** | **COVID status** | **E-Value:**  **Hazard Ratio** | **E-Value:**  **Lower Confidence Limit** | **E-Value:**  **Upper Confidence Limit** |
| --- | --- | --- | --- | --- |
| Composite CV Event | Outpatient | - | - | - |
|  | Inpatient/ICU- | 1.88 | 1.79 | 1.99 |
|  | Inpatient/ICU+ | 3.00 | 2.81 | 3.19 |
| Dysrhythmia | Outpatient | - | - | - |
|  | Inpatient/ICU- | 1.74 | 1.62 | 1.86 |
|  | Inpatient/ICU+ | 2.90 | 2.69 | 3.15 |
| Ischemic heart disease | Outpatient | - | - | - |
|  | Inpatient/ICU- | 1.79 | 1.59 | 1.97 |
|  | Inpatient/ICU+ | 2.56 | 2.26 | 2.87 |
| Other cardiac disorders and myocarditis/pericarditis | Outpatient | - | - | - |
|  | Inpatient/ICU- | 2.66 | 2.41 | 2.94 |
|  | Inpatient/ICU+ | 4.76 | 4.27 | 5.33 |
| Thrombotic disorders | Outpatient | - | - | - |
|  | Inpatient/ICU- | 2.92 | 2.58 | 3.29 |
|  | Inpatient/ICU+ | 4.46 | 3.85 | 5.13 |
| Cerebrovascular accident | Outpatient | - | - | - |
|  | Inpatient/ICU- | 2.06 | 1.86 | 2.26 |
|  | Inpatient/ICU+ | 3.21 | 2.87 | 3.58 |

# Table S10. Risk of alternative categorization of cardiovascular events by COVID-19 level of care setting, 18+

|  | | | | **Weighted 9-month incidence (95% CI)** | | | **Unweighted Hazard Ratio (95% CI)** | | | **Weighted Hazard Ratio (95% CI)** | | |
| --- | --- | --- | --- | --- | --- | --- | --- | --- | --- | --- | --- | --- |
| **Endpoint** | **COVID status** | **Number of events** | **Number of deaths** | **Incidence** | **LCL** | **UCL** | **Hazard Ratio** | **LCL** | **UCL** | **Hazard Ratio** | **LCL** | **UCL** |
| Atherosclerotic CV events | Outpatient | 37036 | 59 | 0.026 | 0.026 | 0.026 | - | - | - | - | - | - |
|  | Inpatient/ICU- | 3053 | 14 | 0.035 | 0.033 | 0.037 | 2.45 | 2.36 | 2.54 | 1.31 | 1.25 | 1.37 |
|  | Inpatient/ICU+ | 1977 | 19 | 0.047 | 0.044 | 0.051 | 3.36 | 3.21 | 3.52 | 1.77 | 1.65 | 1.89 |
| Inflammation CV events | Outpatient | 80377 | 94 | 0.055 | 0.055 | 0.056 | - | - | - | - | - | - |
|  | Inpatient/ICU- | 4428 | 17 | 0.071 | 0.068 | 0.074 | 1.64 | 1.59 | 1.69 | 1.26 | 1.22 | 1.31 |
|  | Inpatient/ICU+ | 2800 | 25 | 0.103 | 0.097 | 0.109 | 2.23 | 2.14 | 2.31 | 1.81 | 1.70 | 1.91 |
| Acute CV events | Outpatient | 26405 | 145 | 0.019 | 0.018 | 0.019 | - | - | - | - | - | - |
|  | Inpatient/ICU- | 2264 | 28 | 0.027 | 0.025 | 0.028 | 2.53 | 2.43 | 2.65 | 1.40 | 1.32 | 1.48 |
|  | Inpatient/ICU+ | 1515 | 31 | 0.037 | 0.034 | 0.040 | 3.59 | 3.41 | 3.78 | 1.90 | 1.77 | 2.05 |
| Chronic CV events | Outpatient | 90822 | 46 | 0.063 | 0.062 | 0.063 | - | - | - | - | - | - |
|  | Inpatient/ICU- | 5251 | 8 | 0.080 | 0.077 | 0.083 | 1.73 | 1.69 | 1.78 | 1.25 | 1.21 | 1.30 |
|  | Inpatient/ICU+ | 3262 | 15 | 0.113 | 0.107 | 0.120 | 2.32 | 2.24 | 2.40 | 1.76 | 1.66 | 1.85 |

Table S11. Risk of cardiovascular events by COVID-19 level of care setting, 18-49

|  | |  | **Weighted 9-month incidence (95% CI)** | | | **Unweighted Hazard Ratio (95% CI)** | | | **Weighted Hazard Ratio (95% CI)** | | |
| --- | --- | --- | --- | --- | --- | --- | --- | --- | --- | --- | --- |
| **Endpoint** | **COVID status** | **Number of events** | **Incidence** | **LCL** | **UCL** | **Hazard Ratio** | **LCL** | **UCL** | **Hazard Ratio** | **LCL** | **UCL** |
| Composite CV Event | Outpatient | 54703 | 0.055 | 0.055 | 0.056 | - | - | - | - | - | - |
|  | Inpatient/ICU- | 2093 | 0.071 | 0.066 | 0.075 | 1.62 | 1.55 | 1.69 | 1.24 | 1.18 | 1.30 |
|  | Inpatient/ICU+ | 1256 | 0.112 | 0.102 | 0.122 | 2.68 | 2.54 | 2.84 | 1.92 | 1.79 | 2.05 |
| Dysrhythmia | Outpatient | 44002 | 0.044 | 0.044 | 0.045 | - | - | - | - | - | - |
|  | Inpatient/ICU- | 1569 | 0.054 | 0.050 | 0.058 | 1.49 | 1.42 | 1.57 | 1.18 | 1.11 | 1.25 |
|  | Inpatient/ICU+ | 911 | 0.085 | 0.077 | 0.095 | 2.36 | 2.21 | 2.52 | 1.79 | 1.66 | 1.93 |
| Ischemic heart disease | Outpatient | 4689 | 0.005 | 0.005 | 0.005 | - | - | - | - | - | - |
|  | Inpatient/ICU- | 220 | 0.007 | 0.005 | 0.008 | 1.93 | 1.68 | 2.21 | 1.34 | 1.14 | 1.57 |
|  | Inpatient/ICU+ | 187 | 0.011 | 0.008 | 0.015 | 4.36 | 3.77 | 5.05 | 2.19 | 1.79 | 2.69 |
| Other cardiac disorders and myocarditis/pericarditis | Outpatient | 3063 | 0.003 | 0.003 | 0.003 | - | - | - | - | - | - |
|  | Inpatient/ICU- | 239 | 0.006 | 0.005 | 0.007 | 3.21 | 2.82 | 3.67 | 1.79 | 1.50 | 2.12 |
|  | Inpatient/ICU+ | 208 | 0.012 | 0.009 | 0.016 | 7.45 | 6.47 | 8.57 | 3.58 | 2.93 | 4.37 |
| Thrombotic disorders | Outpatient | 3399 | 0.003 | 0.003 | 0.004 | - | - | - | - | - | - |
|  | Inpatient/ICU- | 200 | 0.006 | 0.005 | 0.008 | 2.42 | 2.10 | 2.79 | 1.77 | 1.50 | 2.09 |
|  | Inpatient/ICU+ | 146 | 0.011 | 0.008 | 0.015 | 4.69 | 3.98 | 5.54 | 2.95 | 2.39 | 3.64 |
| Cerebrovascular accident | Outpatient | 7287 | 0.007 | 0.007 | 0.007 | - | - | - | - | - | - |
|  | Inpatient/ICU- | 358 | 0.010 | 0.009 | 0.012 | 2.02 | 1.82 | 2.25 | 1.42 | 1.25 | 1.61 |
|  | Inpatient/ICU+ | 254 | 0.017 | 0.013 | 0.021 | 3.83 | 3.38 | 4.34 | 2.26 | 1.92 | 2.66 |

Table S12. Risk of cardiovascular events by COVID-19 level of care setting, 50-64

|  | | | | **Weighted 9-month incidence (95% CI)** | | | **Unweighted Hazard Ratio (95% CI)** | | | **Weighted Hazard Ratio (95% CI)** | | |
| --- | --- | --- | --- | --- | --- | --- | --- | --- | --- | --- | --- | --- |
| **Endpoint** | **COVID status** | **Number of events** | **Number of deaths** | **Incidence** | **LCL** | **UCL** | **Hazard Ratio** | **LCL** | **UCL** | **Hazard Ratio** | **LCL** | **UCL** |
| Composite CV Event | Outpatient | 36490 | 13 | 0.098 | 0.097 | 0.099 | - | - | - | - | - | - |
|  | Inpatient/ICU- | 2544 | 2 | 0.134 | 0.128 | 0.140 | 1.61 | 1.54 | 1.67 | 1.37 | 1.30 | 1.43 |
|  | Inpatient/ICU+ | 1736 | 6 | 0.162 | 0.152 | 0.171 | 1.88 | 1.79 | 1.97 | 1.64 | 1.55 | 1.75 |
| Dysrhythmia | Outpatient | 21969 | 39 | 0.058 | 0.058 | 0.059 | - | - | - | - | - | - |
|  | Inpatient/ICU- | 1422 | 4 | 0.077 | 0.072 | 0.082 | 1.45 | 1.38 | 1.54 | 1.30 | 1.22 | 1.39 |
|  | Inpatient/ICU+ | 1038 | 11 | 0.099 | 0.091 | 0.106 | 1.80 | 1.69 | 1.92 | 1.67 | 1.55 | 1.80 |
| Ischemic heart disease | Outpatient | 9799 | 60 | 0.026 | 0.025 | 0.026 | - | - | - | - | - | - |
|  | Inpatient/ICU- | 675 | 11 | 0.033 | 0.029 | 0.036 | 1.53 | 1.42 | 1.66 | 1.26 | 1.15 | 1.39 |
|  | Inpatient/ICU+ | 456 | 15 | 0.038 | 0.033 | 0.042 | 1.72 | 1.57 | 1.89 | 1.39 | 1.24 | 1.56 |
| Other cardiac disorders and myocarditis/pericarditis | Outpatient | 4190 | 35 | 0.011 | 0.011 | 0.012 | - | - | - | - | - | - |
|  | Inpatient/ICU- | 477 | 6 | 0.020 | 0.018 | 0.022 | 2.54 | 2.31 | 2.79 | 1.71 | 1.53 | 1.92 |
|  | Inpatient/ICU+ | 392 | 11 | 0.029 | 0.025 | 0.033 | 3.50 | 3.15 | 3.88 | 2.45 | 2.16 | 2.77 |
| Thrombotic disorders | Outpatient | 3025 | 60 | 0.008 | 0.008 | 0.008 | - | - | - | - | - | - |
|  | Inpatient/ICU- | 297 | 11 | 0.016 | 0.014 | 0.018 | 2.18 | 1.93 | 2.46 | 1.86 | 1.61 | 2.14 |
|  | Inpatient/ICU+ | 211 | 16 | 0.018 | 0.014 | 0.021 | 2.58 | 2.24 | 2.97 | 2.07 | 1.75 | 2.45 |
| Cerebrovascular accident | Outpatient | 8702 | 59 | 0.022 | 0.022 | 0.023 | - | - | - | - | - | - |
|  | Inpatient/ICU- | 670 | 9 | 0.032 | 0.029 | 0.036 | 1.72 | 1.59 | 1.86 | 1.38 | 1.25 | 1.51 |
|  | Inpatient/ICU+ | 501 | 16 | 0.042 | 0.037 | 0.047 | 2.15 | 1.97 | 2.36 | 1.76 | 1.57 | 1.97 |

Table S13. Risk of cardiovascular events by COVID-19 level of care setting, 65+

|  | | | | **Weighted 9-month incidence (95% CI)** | | | **Unweighted Hazard Ratio (95% CI)** | | | **Weighted Hazard Ratio (95% CI)** | | |
| --- | --- | --- | --- | --- | --- | --- | --- | --- | --- | --- | --- | --- |
| **Endpoint** | **COVID status** | **Number of events** | **Number of deaths** | **Incidence** | **LCL** | **UCL** | **Hazard Ratio** | **LCL** | **UCL** | **Hazard Ratio** | **LCL** | **UCL** |
| Composite CV Event | Outpatient | 16535 | 12 | 0.173 | 0.171 | 0.176 | - | - | - | - | - | - |
|  | Inpatient/ICU- | 1920 | 4 | 0.203 | 0.193 | 0.214 | 1.26 | 1.20 | 1.32 | 1.18 | 1.11 | 1.25 |
|  | Inpatient/ICU+ | 1073 | 3 | 0.233 | 0.216 | 0.250 | 1.39 | 1.31 | 1.48 | 1.36 | 1.26 | 1.48 |
| Dysrhythmia | Outpatient | 7916 | 28 | 0.082 | 0.080 | 0.084 | - | - | - | - | - | - |
|  | Inpatient/ICU- | 908 | 12 | 0.094 | 0.086 | 0.102 | 1.21 | 1.13 | 1.30 | 1.16 | 1.07 | 1.26 |
|  | Inpatient/ICU+ | 509 | 10 | 0.109 | 0.097 | 0.121 | 1.33 | 1.21 | 1.45 | 1.28 | 1.15 | 1.43 |
| Ischemic heart disease | Outpatient | 5351 | 42 | 0.055 | 0.054 | 0.057 | - | - | - | - | - | - |
|  | Inpatient/ICU- | 595 | 16 | 0.060 | 0.054 | 0.066 | 1.17 | 1.07 | 1.27 | 1.06 | 0.96 | 1.17 |
|  | Inpatient/ICU+ | 341 | 12 | 0.070 | 0.060 | 0.079 | 1.30 | 1.17 | 1.45 | 1.22 | 1.06 | 1.39 |
| Other cardiac disorders and myocarditis/pericarditis | Outpatient | 3463 | 27 | 0.036 | 0.034 | 0.037 | - | - | - | - | - | - |
|  | Inpatient/ICU- | 509 | 12 | 0.049 | 0.044 | 0.055 | 1.55 | 1.41 | 1.70 | 1.33 | 1.19 | 1.48 |
|  | Inpatient/ICU+ | 315 | 6 | 0.068 | 0.057 | 0.080 | 1.87 | 1.66 | 2.10 | 1.75 | 1.49 | 2.06 |
| Thrombotic disorders | Outpatient | 1531 | 56 | 0.015 | 0.015 | 0.016 | - | - | - | - | - | - |
|  | Inpatient/ICU- | 255 | 18 | 0.023 | 0.020 | 0.027 | 1.75 | 1.53 | 2.00 | 1.49 | 1.28 | 1.74 |
|  | Inpatient/ICU+ | 149 | 17 | 0.032 | 0.026 | 0.039 | 1.97 | 1.66 | 2.33 | 1.90 | 1.56 | 2.32 |
| Cerebrovascular accident | Outpatient | 5433 | 45 | 0.055 | 0.053 | 0.056 | - | - | - | - | - | - |
|  | Inpatient/ICU- | 644 | 18 | 0.070 | 0.063 | 0.077 | 1.25 | 1.15 | 1.36 | 1.18 | 1.07 | 1.31 |
|  | Inpatient/ICU+ | 358 | 16 | 0.072 | 0.061 | 0.082 | 1.35 | 1.21 | 1.50 | 1.28 | 1.11 | 1.48 |

# Table S14. Risk of cardiovascular events requiring inpatient care by COVID-19 level of care setting, 18-49

|  | |  | **Weighted 9-month incidence (95% CI)** | | | **Unweighted Hazard Ratio (95% CI)** | | | **Weighted Hazard Ratio (95% CI)** | | |
| --- | --- | --- | --- | --- | --- | --- | --- | --- | --- | --- | --- |
| **Endpoint** | **COVID status** | **Number of events** | **Incidence** | **LCL** | **UCL** | **Hazard Ratio** | **LCL** | **UCL** | **Hazard Ratio** | **LCL** | **UCL** |
| Composite CV Event | Outpatient | 7019 | 0.007 | 0.007 | 0.008 | - | - | - | - | - | - |
|  | Inpatient/ICU- | 602 | 0.017 | 0.015 | 0.019 | 3.62 | 3.33 | 3.94 | 2.13 | 1.92 | 2.37 |
|  | Inpatient/ICU+ | 431 | 0.033 | 0.028 | 0.039 | 7.18 | 6.51 | 7.91 | 4.04 | 3.56 | 4.59 |
| Dysrhythmia | Outpatient | 4580 | 0.005 | 0.005 | 0.005 | - | - | - | - | - | - |
|  | Inpatient/ICU- | 435 | 0.012 | 0.010 | 0.014 | 3.98 | 3.61 | 4.39 | 2.39 | 2.11 | 2.71 |
|  | Inpatient/ICU+ | 295 | 0.024 | 0.019 | 0.029 | 7.35 | 6.54 | 8.27 | 4.41 | 3.79 | 5.12 |
| Ischemic heart disease | Outpatient | 1131 | 0.001 | 0.001 | 0.001 | - | - | - | - | - | - |
|  | Inpatient/ICU- | 90 | 0.003 | 0.002 | 0.004 | 3.26 | 2.63 | 4.04 | 2.00 | 1.53 | 2.62 |
|  | Inpatient/ICU+ | 67 | 0.003 | 0.002 | 0.005 | 6.45 | 5.04 | 8.26 | 2.98 | 2.09 | 4.27 |
| Other cardiac disorders and myocarditis/pericarditis | Outpatient | 962 | 0.001 | 0.001 | 0.001 | - | - | - | - | - | - |
|  | Inpatient/ICU- | 116 | 0.002 | 0.002 | 0.003 | 4.98 | 4.11 | 6.04 | 2.34 | 1.79 | 3.07 |
|  | Inpatient/ICU+ | 113 | 0.006 | 0.004 | 0.009 | 12.94 | 10.65 | 15.72 | 5.72 | 4.31 | 7.58 |
| Thrombotic disorders | Outpatient | 945 | 0.001 | 0.001 | 0.001 | - | - | - | - | - | - |
|  | Inpatient/ICU- | 84 | 0.002 | 0.001 | 0.003 | 3.65 | 2.92 | 4.56 | 1.92 | 1.42 | 2.60 |
|  | Inpatient/ICU+ | 70 | 0.005 | 0.003 | 0.008 | 8.07 | 6.33 | 10.29 | 4.71 | 3.43 | 6.46 |
| Cerebrovascular accident | Outpatient | 2209 | 0.002 | 0.002 | 0.002 | - | - | - | - | - | - |
|  | Inpatient/ICU- | 130 | 0.003 | 0.002 | 0.004 | 2.43 | 2.04 | 2.90 | 1.40 | 1.11 | 1.76 |
|  | Inpatient/ICU+ | 122 | 0.007 | 0.005 | 0.010 | 6.10 | 5.08 | 7.32 | 2.93 | 2.26 | 3.79 |

# Table S15. Risk of cardiovascular events requiring inpatient care by COVID-19 level of care setting, 50-64

|  | | | | **Weighted 9-month incidence (95% CI)** | | | **Unweighted Hazard Ratio (95% CI)** | | | **Weighted Hazard Ratio (95% CI)** | | |
| --- | --- | --- | --- | --- | --- | --- | --- | --- | --- | --- | --- | --- |
| **Endpoint** | **COVID status** | **Number of events** | **Number of deaths** | **Incidence** | **LCL** | **UCL** | **Hazard Ratio** | **LCL** | **UCL** | **Hazard Ratio** | **LCL** | **UCL** |
| Composite CV Event | Outpatient | 5678 | 13 | 0.016 | 0.016 | 0.017 | - | - | - | - | - | - |
|  | Inpatient/ICU- | 696 | 2 | 0.032 | 0.029 | 0.035 | 2.82 | 2.61 | 3.05 | 1.90 | 1.72 | 2.08 |
|  | Inpatient/ICU+ | 564 | 6 | 0.048 | 0.043 | 0.053 | 3.91 | 3.58 | 4.27 | 2.87 | 2.58 | 3.20 |
| Dysrhythmia | Outpatient | 3214 | 39 | 0.009 | 0.009 | 0.009 | - | - | - | - | - | - |
|  | Inpatient/ICU- | 380 | 4 | 0.017 | 0.015 | 0.019 | 2.66 | 2.39 | 2.95 | 1.79 | 1.57 | 2.03 |
|  | Inpatient/ICU+ | 336 | 11 | 0.028 | 0.024 | 0.032 | 3.98 | 3.56 | 4.46 | 3.00 | 2.62 | 3.44 |
| Ischemic heart disease | Outpatient | 1939 | 60 | 0.005 | 0.005 | 0.005 | - | - | - | - | - | - |
|  | Inpatient/ICU- | 195 | 11 | 0.007 | 0.006 | 0.009 | 2.24 | 1.93 | 2.59 | 1.45 | 1.21 | 1.73 |
|  | Inpatient/ICU+ | 162 | 15 | 0.012 | 0.009 | 0.014 | 3.09 | 2.63 | 3.63 | 2.08 | 1.70 | 2.53 |
| Other cardiac disorders and myocarditis/pericarditis | Outpatient | 1393 | 35 | 0.004 | 0.004 | 0.004 | - | - | - | - | - | - |
|  | Inpatient/ICU- | 196 | 6 | 0.007 | 0.006 | 0.008 | 3.14 | 2.70 | 3.64 | 1.74 | 1.46 | 2.08 |
|  | Inpatient/ICU+ | 194 | 11 | 0.014 | 0.011 | 0.016 | 5.19 | 4.46 | 6.04 | 3.42 | 2.87 | 4.08 |
| Thrombotic disorders | Outpatient | 900 | 60 | 0.002 | 0.002 | 0.003 | - | - | - | - | - | - |
|  | Inpatient/ICU- | 134 | 11 | 0.006 | 0.004 | 0.007 | 3.29 | 2.75 | 3.95 | 2.20 | 1.77 | 2.75 |
|  | Inpatient/ICU+ | 100 | 16 | 0.008 | 0.006 | 0.010 | 4.09 | 3.33 | 5.04 | 3.17 | 2.47 | 4.06 |
| Cerebrovascular accident | Outpatient | 2316 | 59 | 0.006 | 0.006 | 0.006 | - | - | - | - | - | - |
|  | Inpatient/ICU- | 266 | 9 | 0.013 | 0.011 | 0.015 | 2.56 | 2.26 | 2.91 | 1.94 | 1.67 | 2.26 |
|  | Inpatient/ICU+ | 220 | 16 | 0.018 | 0.014 | 0.021 | 3.56 | 3.10 | 4.09 | 2.63 | 2.23 | 3.11 |

# Table S16. Risk of cardiovascular events requiring inpatient care by COVID-19 level of care setting, 65+

|  | | | | **Weighted 9-month incidence (95% CI)** | | | **Unweighted Hazard Ratio (95% CI)** | | | **Weighted Hazard Ratio (95% CI)** | | |
| --- | --- | --- | --- | --- | --- | --- | --- | --- | --- | --- | --- | --- |
| **Endpoint** | **COVID status** | **Number of events** | **Number of deaths** | **Incidence** | **LCL** | **UCL** | **Hazard Ratio** | **LCL** | **UCL** | **Hazard Ratio** | **LCL** | **UCL** |
| Composite CV Event | Outpatient | 3425 | 12 | 0.039 | 0.037 | 0.040 | - | - | - | - | - | - |
|  | Inpatient/ICU- | 598 | 4 | 0.064 | 0.057 | 0.070 | 1.89 | 1.73 | 2.06 | 1.60 | 1.44 | 1.78 |
|  | Inpatient/ICU+ | 380 | 3 | 0.087 | 0.076 | 0.098 | 2.37 | 2.13 | 2.64 | 2.23 | 1.95 | 2.55 |
| Dysrhythmia | Outpatient | 2047 | 28 | 0.022 | 0.021 | 0.023 | - | - | - | - | - | - |
|  | Inpatient/ICU- | 327 | 12 | 0.033 | 0.028 | 0.038 | 1.69 | 1.50 | 1.90 | 1.45 | 1.25 | 1.67 |
|  | Inpatient/ICU+ | 213 | 10 | 0.047 | 0.038 | 0.056 | 2.15 | 1.86 | 2.47 | 2.07 | 1.72 | 2.49 |
| Ischemic heart disease | Outpatient | 1331 | 42 | 0.014 | 0.013 | 0.015 | - | - | - | - | - | - |
|  | Inpatient/ICU- | 211 | 16 | 0.020 | 0.016 | 0.023 | 1.67 | 1.44 | 1.93 | 1.44 | 1.21 | 1.72 |
|  | Inpatient/ICU+ | 121 | 12 | 0.028 | 0.021 | 0.035 | 1.85 | 1.54 | 2.23 | 1.84 | 1.45 | 2.34 |
| Other cardiac disorders and myocarditis/pericarditis | Outpatient | 1130 | 27 | 0.012 | 0.011 | 0.013 | - | - | - | - | - | - |
|  | Inpatient/ICU- | 213 | 12 | 0.020 | 0.017 | 0.024 | 1.99 | 1.72 | 2.31 | 1.61 | 1.36 | 1.92 |
|  | Inpatient/ICU+ | 148 | 6 | 0.030 | 0.023 | 0.036 | 2.69 | 2.27 | 3.20 | 2.22 | 1.79 | 2.75 |
| Thrombotic disorders | Outpatient | 571 | 56 | 0.006 | 0.005 | 0.006 | - | - | - | - | - | - |
|  | Inpatient/ICU- | 127 | 18 | 0.011 | 0.009 | 0.013 | 2.33 | 1.92 | 2.83 | 1.80 | 1.45 | 2.24 |
|  | Inpatient/ICU+ | 82 | 17 | 0.018 | 0.013 | 0.023 | 2.91 | 2.31 | 3.68 | 2.77 | 2.11 | 3.64 |
| Cerebrovascular accident | Outpatient | 1455 | 45 | 0.015 | 0.014 | 0.016 | - | - | - | - | - | - |
|  | Inpatient/ICU- | 231 | 18 | 0.026 | 0.021 | 0.030 | 1.67 | 1.46 | 1.92 | 1.53 | 1.29 | 1.80 |
|  | Inpatient/ICU+ | 141 | 16 | 0.029 | 0.023 | 0.036 | 1.98 | 1.67 | 2.36 | 1.83 | 1.47 | 2.27 |

Figure S1. Risk of individual cardiovascular events by COVID-19 level of care setting, 18+


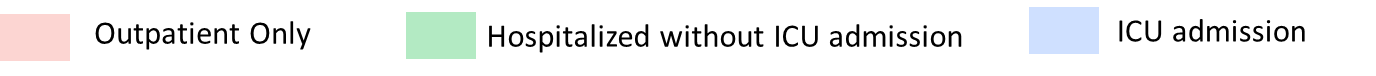


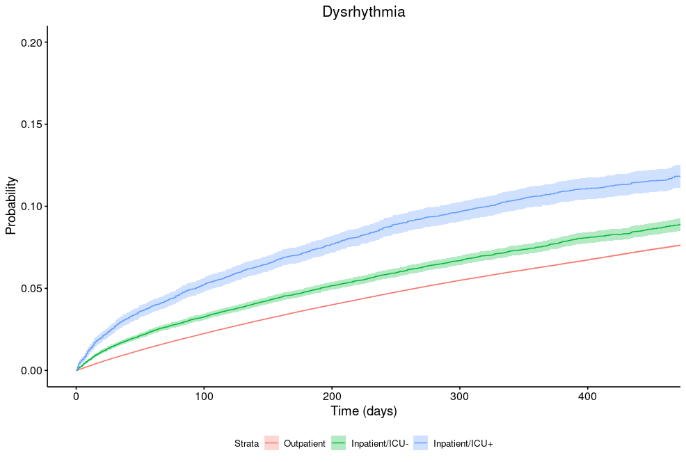

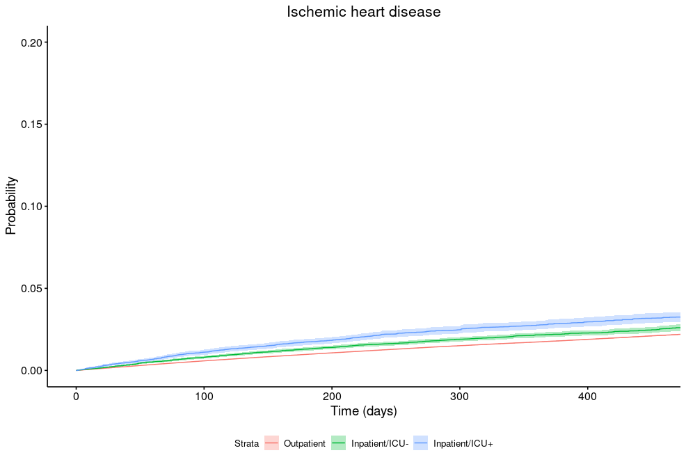


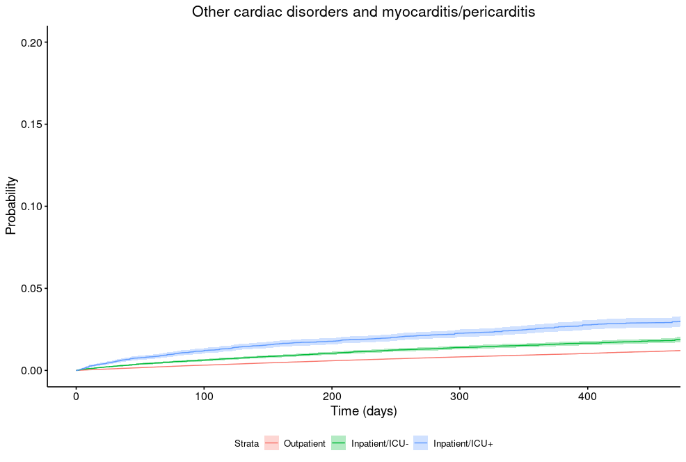

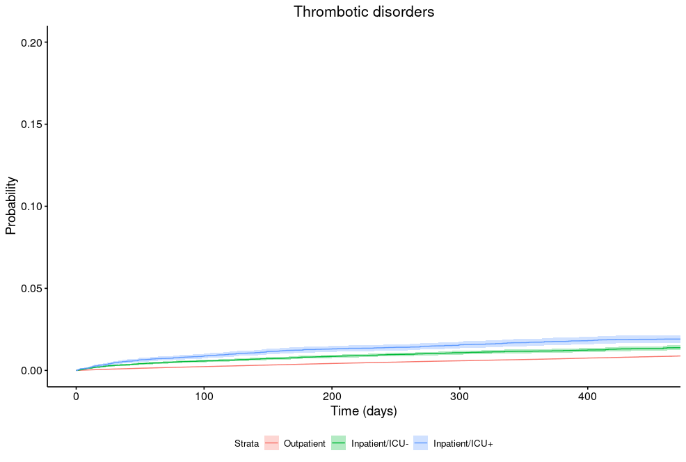


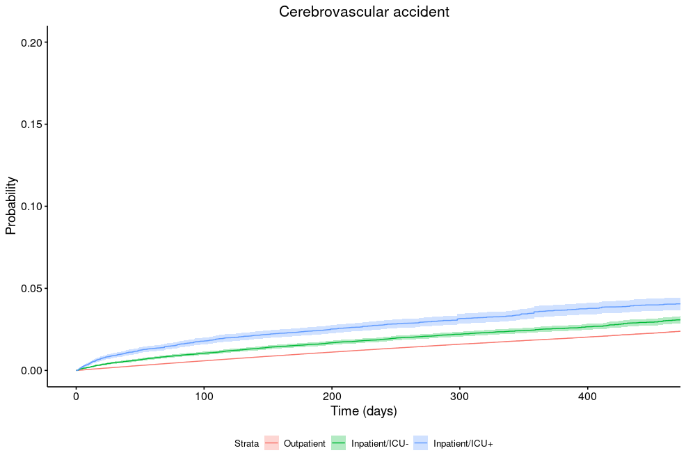


# Table S17. Number of patients with COVID treatment and specific outcome codes

|  | **Number** | **%** |
| --- | --- | --- |
| Total number of patients | **1,357,518** | **100.0** |
| Drugs observed during 30-day assessment period | | |
| Remdesivir | 3,939 | 0.29 |
| Dexamethasone | 62,707 | 4.62 |
| Monoclonal antibodies | 12,572 | 0.92 |
| Specific outcomes during follow-up | | |
| D65: disseminated intravascular coagulation | 150 | 0.01 |
| R00.X: abnormalities of heartbeat | 66,223 | 4.88 |
